# ‘Challenging but ultimately rewarding’ — lived experiences of Deep End Northern Ireland GPs: a qualitative study

**DOI:** 10.3399/BJGP.2024.0167

**Published:** 2024-10-29

**Authors:** Daniel Butler, Diarmuid O’Donovan, Jenny Johnston, Nigel Hart

**Affiliations:** School of Medicine, Dentistry and Biomedical Sciences, Queen’s University Belfast, Belfast.; Queen’s University Belfast; honorary consultant in public health, Public Health Agency, Belfast.; School of Medicine, Dentistry and Biomedical Sciences, Queen’s University Belfast, Belfast.; School of Medicine, Dentistry and Biomedical Sciences, Queen’s University Belfast, Belfast.

**Keywords:** family practice, general practice, health inequities, healthcare disparities, qualitative research, mental health

## Abstract

**Background:**

Living in socioeconomically deprived areas is associated with shorter lives and worse health. GPs working in these areas face additional challenges compared with those in more affluent locations.

**Aim:**

To establish GPs’ motivation for working in these areas, to discover the challenges that GPs face, and to gain insights from GPs on potential improvements and changes.

**Design and setting:**

An interpretative phenomenological analysis was undertaken of GPs’ lived experiences of working in the most socioeconomically deprived practices in Northern Ireland (NI), which is the most deprived country within the UK.

**Method:**

Interviews were carried out with nine GPs to find out the challenges facing them, why they work in a Deep End area, and what suggestions, ideas, and solutions they have to improve patient care and GP experience at NI’s Deep End.

**Results:**

The challenges related to wider health service failures including the increased demand on GPs and feelings of powerlessness. Patient population challenges included ‘missingness’, late or crisis presentations, alongside the clinical difficulties of a highly ‘medicalised’ patient population, as well as the high prevalence of mental health problems. However, GPs choose to work in Deep End areas because the environments were seen as clinically stimulating and rewarding, as well as giving them feelings of belonging and fulfilling a duty to ‘their’ area. Improvements focused on providing more flexible access, increased mental health provision, and future training and recruitment, particularly around widening participation in medical school.

**Conclusion:**

Improving the environmental conditions, empowering individuals, and investing in communities are essential factors to achieving health. The current model of providing reactionary acute care is leading to GPs experiencing powerlessness and feelings of helplessness at the Deep End.

## Introduction

General practice in areas of socioeconomic deprivation faces additional challenges. Individuals in these areas live shorter lives in worse health.^1^^,^^2^ GPs face larger patient lists, increased demand, higher physical and mental health comorbidity, and shorter consultations, with current funding not matching clinical need.^3^^–^7 Alongside the demand versus resource mismatch^7^^,^^8^ are the large number of social presentations faced by Deep End GPs,^7^ concerns around the increasing roll-out of digital and remote innovations in primary care,^9^^,^^10^ the significant unrecognised ‘hidden’ workload,^8^ and the current workforce crisis.^11^^–^13 The Deep End projects, over the past 15 years, have been networks advocating for and working towards closing the health inequalities gap; however, Northern Ireland (NI) has lagged behind, despite being the most deprived of the UK nations.^14^

There are unique challenges to health care in NI relating to the ‘Troubles’, a recent civil conflict, spanning three decades, resulting in more than 3500 deaths, 34 000 shootings, and 14 000 bombings.^15^^,^^16^ At time of publication, NI has the longest secondary care waiting lists^17^^,^^18^ and highest suicide rate in the UK.^19^ It also has a political system that has been stalled for more time than it has functioned over the past 7 years. Within the UK context, NI can be seen as the Deep End of the Deep End. Unlike the rest of the UK, general practice-level socioeconomic data are not freely available, and therefore the unique challenges and needs of GPs at the Deep End are largely unseen and unrecognised. This study specifically focuses on the GPs’ perspectives in NI.

This study aimed to find out the following:

- the specific challenges facing Deep End GPs in NI;
- why GPs work in a Deep End area (the positive, protective aspects); and
- suggestions, ideas, and solutions to improve patient care and GP experience at NI’s Deep End.

## Method

Phenomenology was the theoretical perspective used, given it explores both what was experienced and how it was experienced,^20^^,^^21^ specifically interpretative phenomenological analysis (IPA). IPA takes an interpretative approach to the data, with the researcher(s) making sense of the participants considering their experiences.^22^ The participants were GPs working at the Deep End, which is defined as general practices with more than half the patient list living in the most socioeconomically deprived quintile as described in the authors’ previous work.^23^^,^^24^ The structure of IPA nurtures the reflexivity, allowing for identification, description, analysis, organisation, and reporting of themes that are drawn from the data.^25^^,^^26^ Semi-structured interviews were used for data capture, which is regarded as the exemplary method for data collection in IPA.^25^ The interview schedule was developed by the research team through consultation with GP representatives from the most deprived GP Federations in NI. A purposive sampling approach was taken to maximise variation with an email invitation to potential participants from the list of general practices operating in the most socioeconomically deprived areas of NI. See Box 1 for inclusion and exclusion criteria. This approach has previously been adopted in Scotland for similar work.^27^ The email invite and participant information sheet are included within Supplementary Information S1 and S2.

**Table:** How this fits in

GPs working in the highest need, socioeconomically deprived areas — the Deep End — face additional challenges compared with those in more affluent locations. This study looks at the Northern Ireland (NI) context and explores why, despite the challenges, GPs choose to work in these areas. The main issues relate to wider healthcare failings and the challenges of patient populations, some of whom generally frequently use (‘medicalised’ group) and those who underuse (‘missingness’ group) health services. GPs tend to relate to Deep End areas, either owing to personal connections or feelings of duty and social responsibility. No amount of funding focused on general practice will ‘solve’ the issues; instead, a far greater holistic approach, improving the physical conditions in which people are born, live, and work in, is needed.

**Box 1.** Inclusion and exclusion criteria

**Inclusion criteria**	**Exclusion criteria**
Must be fully qualified with a Certificate of Completion of Training (CCT)	GP registrars
Willingness to communicate in English language	GPs who do not work in areas of significant socioeconomic deprivation
Willingness to sign the informed consent form and partake in a one-to-one online interview	

The interviews took place remotely using Microsoft Teams, enabling GP engagement uninhibited by location. Interviews were conducted and manually transcribed by the first author, a GP, who had undergone formal qualitative research training as part of their postgraduate studies. Data analysis followed the IPA steps as directed by Smith *et al*.^25^^,^^28^^,^^29^ The steps were as follows: data familiarisation; note-making on interesting parts of the data; theme identification from notes (see Supplementary Information S3 and S4 for the interview schedule and example interview with notes and themes);^25^^,^^28^ and a coding framework iteratively developed in NVivo (version 20) for each participant and transcript. The first author completed these steps for all participants; the other members of the research team reviewed transcripts, re-coded, and were part of the development of the coding frameworks. The number of interviews was not pre-set, and interviews would stop once the research team felt theoretical sufficiency had been reached within the data.

## Results

### Descriptive results

Forty-five practices were identified as Deep End. Nine GPs, each from different practices, were interviewed. Recordings lasted between 25 and 60 minutes with a mean length of 38 minutes. Initial data collection started in November 2021, then resumed in March–April 2023 owing to the lead author’s career break. The descriptive details of GP participants can be seen in Table 1.

**Table 1.** Descriptive details of GP participants

**Participant number**	**Gender**	**Position**	**Interview length, mins**	**Experience as GP, years**	**Approximate size of practice**	**Proportion of the patient list in the most deprived quintile, %**	**Urban or rural practice**	**Geographical GP Federation**
1	Female	Partner (5 sessions) and portfolio role with local addictions team	27	10	5000 patients	68	Urban	North Belfast
2	Female	Partner (7 sessions)	43	12	7800 patients	59	Rural	Derry and Strabane
3	Male	Salaried (4 sessions) and sessional (2 sessions)	35	1	5200 patients	68	Urban	North Belfast
4	Female	Sessional (8 sessions)	37	1.5	3700 patients	66	Urban	West Belfast
5	Male	Partner (8 sessions)	41	23	8000 patients	63	Urban	North Belfast
6	Male	Partner (8 sessions)	37	9	4200 patients	93	Rural	Newry and District
7	Female	Partner (7 sessions) and portfolio roles	25	16	10 000 patients	62	Urban	West Belfast
8	Female	Partner (9 sessions)	60	20	10 800 patients	55	Urban	Derry and Strabane
9	Female	Salaried (6 sessions)	37	5	2500 patients	71	Urban	North Belfast

### Narrative results

See Box 2 for a list of the overarching themes and sub-themes related to the research objectives, which are discussed below.

**Box 2.** A list of the overarching themes and sub-themes related to the research objectives

**Research objective:** Establishing the challenges facing Deep End GPs in Northern Ireland	
**Themes**	**Sub-themes**
Healthcare system challenges	Unprecedented increase in demand since COVID-19 pandemic ‘Revolving door’ owing to stretched and absent secondary care services Long waiting lists for secondary care
Challenges related to the patient population	‘Missingness’, non-attenders, or hard-to-reach Crisis or late presentations Low health expectations and generational poor health within communities ‘Medicalisation’ and frequent service users Social problems and the powerlessness as a GP to change the situation De-prescribing pressures Reliance on external support
Challenges related to the social, geographical, and historical context	Rural stigma The Troubles and related implications; the intergenerational trauma, mental health, and ongoing implications to GP workload
**Research objective:** Why work in a Deep End area? Exploring the positive, protective aspects	
**Themes**	**Sub-themes**
Personally rewarding and clinically stimulating	Personal satisfaction Clinically stimulating High index of suspicion, higher prevalence of pathology
Feelings of duty, responsibility, and belonging to an area	Identifying with an area or population Growing up in a specific Deep End area Feelings of social responsibility and duty
**Research objective:** From GPs’ experiences, what are their suggestions, ideas, and possible solutions to improve patient care and GP experience at the Deep End?	
**Themes**	**Sub-themes**
Suggestions, ideas, and possible solutions	Flexible, drop-in style primary healthcare provision Focus on GP training and recruitment in socioeconomically deprived areas Mental health service provision Increased community and social support

### Establishing the challenges facing Deep End GPs in NI

#### Healthcare system challenges

Participants reported an unprecedented increase in demand since the COVID-19 pandemic, with negative experiences of the insurmountable workload, leading to feelings of burnout. This has been worsened by the reported ‘revolving door’ experience within primary care, defined as individuals presenting multiple times with the same issues and *‘recirculating’* (Participant [P]5) within general practice while on hospital waiting lists. The problem is exacerbated in socioeconomically deprived populations where participants report that private health care is usually financially inaccessible:

*‘… demand is through the roof … I could put on ten doctors on every day and I wouldn’t cope with the demand … On top of that, the extremely long waiting lists for secondary care … patients are presenting again and again and again, the same problems, chronic problems that we’re not able to manage and things definitely deteriorated in all chronic diseases.’*

(P7)

*‘I remember someone who was on an urgent wait for urology, for about six years. And you know, there’s nothing that we can do, but they’re still coming and contacting us regularly and we’re having to chase and write letters and yes whereas if they were able to avail of private they would have honestly gotten sorted and dealt with much quicker and had a better patient experience.’*

(P9)

#### Challenges related to the patient population

The GP participants predominantly described the following two groups of patients in the areas they serve: those who underuse services, the ‘missingness’ group; and those who frequently contact the practices, the ‘medicalised’ group:

*‘… deprived people either overuse the service or don’t use it at all.’*

(P8)

The theme of underuse or delayed presentation to general practice was thought to be related to an individual’s acceptance of poor health. Shorter healthy life expectancy is seen as not only the norm, but also an expectation, owing to patients’ personal experiences and social environments:

*‘I think people in areas of social deprivation tend to have a lower expectation of their own health, and so just accept chronic disease a lot more and expect chronic disease in their lives.’*

(P1)

*‘… it’s a population where they have an expectation of poor health almost. They seem to feel that they shouldn’t be able to walk the length themselves at sixty. This is perfectly normal for a significant number of the people … ’*

(P5)

This was thought to result in patients presenting later with more advanced disease:

*‘… an A&E* [accident and emergency] *consultant said to me when your patients come in to me, they’re properly sick.’*

(P6)

*‘They’re just I call PRN life. You know … ’*

(P8)

(PRN is defined as a commonly used medical term for medication when it is to be used ‘when required’ rather than on a regular basis.)

*‘I saw a fella today. He’s in his thirties who’s been on drugs, who’s type one diabetic. Who has end-stage renal failure in their early thirties, but won’t turn up for his dialysis appointments. Do you know what I mean? And even though he knows it’s life-threatening complications … and this is you know just the norm in areas like this.’*

(P9)

General practice can be the first place that people turn for help and support, and as a result GPs feel tasked to deal with multiple social problems, describing this as the ‘medicalisation’ of life problems. A perceived lack of alternative services can lead to societal and environmental difficulties presenting for help in general practice, leading to feelings of powerlessness and helplessness among both doctors and patients:

*‘I do feel patients speak to you a lot about their social issues. Which as a GP you can’t really do anything about, so certainly … there are times where I think I feel really helpless …* [these] *problems that aren’t necessarily in your medical remit but are more social.’*

(P4)

*‘… more and more, everything seems to be a GP problem, with the GP expected to fix it, and this idea of normal life problems suddenly it’s a problem that needs fixed … see your GP about that. Like a pill for every ill almost.’*

(P7)

Prescribing and de-prescribing were challenging experiences for GPs to navigate, with feelings of frustration, particularly around local Department of Health prescribing reports:

*‘… there’s the expectation that medication is going to help … it’s very difficult saying no ‘cause I think they feel as if you’re rejecting them, and I’m not offering them help in the way that they think they need it.’*

(P1)

*‘I know prescribing an opioid medication isn’t on the NICE* [National Institute for Health and Care Excellence] *guidance. But then whenever you work somewhere like this, you realise, well, this is real life and sometimes this is what happens and these patients are prescribed these medications …* [Trauma and adversity] *Doesn’t fit into the NICE guidelines. And that’s a real common theme.’*

(P4)

#### Challenges related to the social, geographical, and historical context

Rural Deep End GPs described increased complexity around perceived stigma and non-attenders, owing to smaller, rural populations, and while there is less pressure to prescribe controlled drugs, the GPs acknowledge that dependency problems are more hidden:

*‘… despite being an area of huge conflict, lots of bombings, the entire town centre being destroyed. We we’ve still managed, you know, too not prescribed addictive substances. Now that’s all very well. Our patients are still using them. They’re getting them from somewhere, but not, they’re not, getting them on prescription.’*

(P2)

*‘I think there is a drug issue in the area, but it’s not really coming to our doorstep. And I think it’s the stigma of the local country GP surgery … but, like everywhere else, there is a drug issue, but we don’t see it.’*

(P6)

Many participants raised the intergenerational trauma in the lives of their patients linked to the ‘Troubles’. Twenty-five years after the Good Friday Agreement (seen as a key milestone in the peace process), the ongoing implications are experienced frequently. For the younger generation this takes the form of substance misuse and mental health problems, as well as the influence and intimidation by active paramilitary groups, unique to NI:

*‘I think there’s a lot more we’re seeing now of the next generation, who maybe haven’t directly witnessed anything … and we’re seeing more the consequences of them growing up in an unstable childhood environment. And I think that brings a different mental illness that we’re definitely seeing a lot more now through the use of street drugs and alcohol.’*

(P1)

*‘… there’s such huge issues with mental health problems and multifactorial reasons for that, but I think a lot and can be related back to the “Troubles”.’*

(P3)

*‘I can think of two patients in the past year who received paramilitary beatings and they rang to speak to the GP before … asking for diazepam in anticipation of these organised beatings, which is definitely a shock to me … I suggested speaking to the police you know. That’s not an option for them. Or they perceive it’s not … I’m not really sure how I manage it, but I know that I don’t need to ask about paramilitary involvement because patients will just tell me.’*

(P4)

*‘When I first started working here, somebody phoned and said they’re under death threats and I got anxious and worried. Not now. Really badly it’s become normalised … all these bad things suddenly become normalised. I suppose as a GP it’s just part and parcel of what I do every single day and it’s* [the “Troubles” legacy] *still very prevalent and prominent.’*

(P7)

### Why work in a Deep End area? Exploring the positive, protective aspects

#### Personally rewarding and clinically stimulating

Participants reported experiences of personal satisfaction working in socioeconomically deprived areas expressing regard for the patient–doctor interactions, as well as the variety of clinical problems that present:

*‘Yeah, the enormous pathology … There’s a wealth of clinical experience to be gained …* [it’s] *challenging, fulfilling but ultimately very rewarding career, working in a in an area of social deprivation.’*

(P2)

*‘There are easier ways for me to make a living and I’m still here and I actually became a partner. I do it because I love the job. For me, this is proper medicine. This is why I trained to be a GP.’*

(P7)

#### Feelings of duty, responsibility, and belonging to an area

Some GPs identified with their patient population and expressed a commitment to serve the communities they themselves came from:

*‘We were kind of born and raised in the area, so we’re home-grown and we knew a lot of the people already. So that’s why we came back here.’*

(P6)

*‘So this is my community. This is where I grew up. These are my people. And you know to certain extent I do feel that this is why I became a doctor. This is why I became a GP to help this cohort of patients.’*

(P7)

*‘I come from a very working-class background … it’s just really interesting to have that extra layer of lived experience myself.’*

(P8)

Other participants described feelings of justice and a desire to meet the needs of those who need it most:

*‘Yeah, everyone deserves the NHS help and support, but the most deprived, I think, deserve it the most.’*

(P3)

*‘I trained for this very reason. These are the patients that need us most. And I wouldn’t want to work anywhere else.’*

(P7)

### From GPs’ experiences, what are their suggestions, ideas, and possible solutions to improve patient care and GP experience at the Deep End?

#### Flexible, drop-in style primary healthcare provision

Complex social situations were thought to present challenges within current systems. Rethinking booking and creating extra capacity and flexibility in daily access allows practices to respond when someone engages with the healthcare services in an *ad-hoc* way:

*‘I do think they* [patients] *find it difficult to engage with what’s offered in the way it’s offered, so I think they would be able to engage far more in a drop-in whenever suits … as opposed to multiple DNA* [did not attend] *appointments.’*

(P1)

*‘I started realising that with deprivation there was a certain layer of patient that needed more. Either they were too sick to fight the system and fight for an appointment. They were too complex. So we had developed a protocol in the practice called the yellow bubble … They will have a better level of access. It’s not that onerous and it actually, the irony is, the more you give sick people. The better the job is, the more you do it.’*

(P8)

#### Focus of GP training and recruitment in socioeconomically deprived areas

Improving recruitment and retention of GPs at the Deep End was viewed as very important to address. Ideas centred around incentivised training, especially in deprived rural locations, and highlighting the specific registrar opportunities:

*‘I think everybody should work in a deprived area, to see what it’s like and to get a flavour of actually what some people are going through and what their lives are like … The summary is in some ways it* [training in a deprived practice] *equips you better for life as a post-CCT GP.’*

(P4)

*‘I live and breathe this and this is what I trained to do. But it’s not easy. And I think what we need to do is get this into the training programme … start training and getting people through these areas. And actually, you know, start recruiting people to actually do this type of medicine and actually embrace it and not be afraid of it.’*

(P7)

#### Mental health service provision

The volume of mental health presentations and the lack of available services for patients was frequently raised. Participants suggested that increasing mental health service provision and access in areas of socioeconomic deprivation would improve patient care and GP experience:

*‘We’ve poor access to mental health services, we’re recirculating problems all the time.’*

(P5)

#### Increased community and social support

Finally, when asked about future investment, participants focused on upstream societal issues: tackling the social causes. Participants consistently raised areas linked to the social determinants of health as a focus to improve health and wellbeing:

*‘*[Funding] *needs to go back out into the community … Rather than invest money in diabetes or COPD* [chronic obstructive pulmonary disease]*, that for me is not the prevention. It’s actually education, the health literacy, and obviously they all need better housing and better education … ’*

(P7)

## Discussion

### Summary

This study explored the following three areas: the specific challenges facing Deep End GPs in NI; the reasons why GPs work in a Deep End area (the positive, protective aspects); and the suggestions, ideas, and solutions from Deep End GPs to improve patient care and GP experience at NI’s Deep End.

The main challenges facing GPs were the overwhelming patient demand, the ‘revolving door’, or recirculation of repeat presentations owing to overstretched secondary care services and excessive waiting lists, disproportionately impacting patients with the least financial means. Patient population challenges related to how people use general practice, including frequency of crisis presentations and a perceived pressure to prescribe. The social, geographical, and historical context of the area, particularly around the impact and legacy of the ‘Troubles’ and intergenerational trauma, provide additional complexity to the GP’s caseload.

Despite the challenges, the participants did report positive and protective factors that encouraged them to continue working in Deep End areas. These were the personal job satisfaction and the feeling of making a difference, alongside the clinically stimulating type of work. Personal feelings of belonging, duty, and connection with a geographical area were protective and motivational.

Deep End GPs, from their experiences, raised areas for improvement including flexible, drop-in style primary healthcare access, focused GP training and recruitment in socioeconomically deprived areas, increased mental health service provision, and increased investment in community and social support.

### Strengths and limitations

The strengths of this work are the NI focus, the post-conflict society context it resonates with, as well as the incorporation of both urban and rural Deep End practices not commonly seen in Deep End literature. The sample size, while small, is in keeping with phenomenological studies.^29^ Nine GPs, with a range of experience from 1–23 years, represented 20% (*n* = 9/45) of Deep End practices. Given the work pressures, a 20% representation of Deep End practices was thought reasonable. This was further strengthened by interviews eight and nine not raising new themes and data fitting within the coding framework, which suggested theoretical sufficiency had been met through these nine interviews. The data collection process was strengthened by the interview schedule being developed by the whole research team, using input from stakeholders within the four most socioeconomically deprived GP Federations, with the consistency of interview approach afforded by a single researcher (the first author). This semi-structured approach allowed for a richness and depth to the data, which are required when exploring GPs’ lived experiences and perceptions. Rigour was achieved by the methodological approach set out by Smith *et al* for IPA,^22^^,^^25^^,^^28^^,^^29^ including the research team being involved in transcript coding and development of the coding framework.

Following the first three interviews, preliminary results were shared with academic GP colleagues at a national conference. The feedback and questions led to an amendment in the interview schedule with the addition of a ‘silver bullet’ or open cheque-book question around potential improvements.

The work is limited by the time gap between interview collections, which may have implications on the results and data, meaning different pressures were facing general practice at the times of data collection. However, it has given the results more depth, moving beyond the immediate COVID-19 implications, to providing more transferable data looking at the post-COVID-19 transition period. The research team tried to mitigate the time gap between data collection by adding additional steps to strengthen the rigour of the work, with individual review of coded transcripts, and subsequent research team discussion. The lead researcher, who conducted the interviews, was known to three of the interviewees prior, which, given the relatively small size of NI, is to be expected. While the interview guide and semi-structured nature helped ensure consistency, these prior relationships could have impacted the collected data. Finally, as described in the introduction, the context of NI is distinctive compared with the rest of the UK, and therefore application of the results to UK-wide practice should be approached with caution.

### Comparison with existing literature

The distinction between individuals being perceived as ‘overusing’ or ‘underusing’ GP services in this study is consistently seen in Deep End literature. ‘Overusing’ or medicalisation is demonstrated by the high social presentations reported at the Deep End,^7^ the high unrecognised and under-resourced workload,^8^ as well as long waiting lists resulting in reliance on primary care for medication and social supports.^30^ ‘Underuse’ is similar to themes around ‘missingness’, described by Lindsay *et al* as the *‘repeated tendency not to take up offers of care, such that it has a negative impact on the person and their life chances.’* 31^,^^32^ The theme of avoidance has previously been described in similar Deep End populations.^33^^,^^34^ Both avoidance and missingness have resulted in worse morbidity and mortality outcomes.^35^

While the trauma of ‘the Troubles’ is well documented,^16^^,^^19^^,^^36^^–^38 this study describes the ongoing implications in general practice, namely the legacy of trauma on health and the intergenerational link. The epigenetic risks associated with the ‘Troubles’ have been described in the Commission for Victims and Survivors report, *Towards a Better Future: the Trans-generational Impact of the Troubles on Mental Health*,^39^ and the transgenerational epigenetic changes offer a theory for the increased physical comorbidity and early mortality experienced by GPs at the Deep End. Furthermore, ‘learnt helplessness’ theories, whereby persistent adversity creates negative behaviour change, acceptance, and powerlessness,^40^ appear consistent with the GPs’ experiences. The consequences of growing up in an area seen as a ‘Troubles’ hotspot anecdotally correlates with increased generational trauma and the role of adverse childhood experiences (ACEs).^41^ The intergenerational legacy of this trauma is particularly evidenced by the mental health prevalence, the overmedicalisation of some patients, and the perceived disempowerment or acceptance of poor health by others.

Regarding positive and protective factors, the rewarding aspect of the work and potential to make a positive difference in an individual’s life has previously been documented as providing powerful motivation and resilience to Deep End GPs.^8^ Participants identifying with and feeling a connection to an area were seen as motivational and protective, supporting the development of initiatives that seek to widen participation in medical schools as one way to reduce inequities within health care.^42^

Once qualified, the experience gained working in particular clinical settings influences future career choices.^43^^,^^44^ This study supports this finding, with many of the participants having completed their postgraduate training in areas of socioeconomic deprivation, suggesting one way to increase workforce is through focused postgraduate training in Deep End areas, in keeping with existing literature.^23^^,^^42^

### Implications for practice

Regarding learnt helplessness and the social determinants of health, the wider social and environmental context of Deep End areas can lead to GPs experiencing powerlessness, disengagement, and a learnt helplessness. Issues such as material poverty, poor housing conditions, low educational attainment, lack of accessible health services, generational poor health, and intergenerational trauma meant that the wider social determinants of health were the focus of GPs when making sense of their experiences and discussing possible solutions and improvements. Achieving ‘health’ by empowering people and communities requires focused attention on the wider social determinants of health. Failure to do so will maintain the focus on reactionary acute care, leading to greater pressure on primary healthcare services, GPs, and patients.

Regarding mental health prevalence and provision, the high mental health prevalence within NI is influenced by the recent civil conflict, generational trauma, ongoing adversity, and high prevalence of ACEs. The findings are transferable to societies and populations that have experienced recent trauma. The study highlights a clear focus for healthcare policymakers around mental health provision for those living in Deep End areas, where the mental health needs of individuals are significant and complex.

General practice policy should incorporate the ‘Deep End’. The medical complexity of the patient populations in Deep End practices, from those who present late or in crisis, to those who are highly medicalised, means that health policy must be adapted to the community-specific context to be successful. General practice policy or initiatives need to consider the specific challenges faced by colleagues in Deep End areas, not doing so will likely increase disillusionment, burnout, and negatively impact morale within the Deep End workforce.

Regarding GP training and medical recruitment, with NI being the most socioeconomically deprived part of the UK and the high number of Deep End practices involved in training,^24^^,^^45^ considering measures to increase GP registrars’ exposure to Deep End areas for at least one placement on their training journey seems feasible. This would have educational benefit for postgraduate doctors, while increasing personal confidence, likely having positive downstream implications on early career recruitment in Deep End areas.

Finally, identifying with the patient population is a strong protective and positive factor to working as a Deep End GP. Widening participation into medical school has long been described as a measure to reduce health inequalities. This study shows how it is also a positive and protective factor in becoming a GP in a socioeconomically deprived area.

In conclusion, despite the challenges raised by GPs at the Deep End, there are strong positive, protective, and supportive aspects to the work. Highlighting these, and building on them, is essential to develop the GP workforce and improve care for those in society who need it most.

## Data

The dataset (full transcripts) are not publicly available currently.
